# Differential Effect of Abusing Substances Alone or in Combination in Systemic Inflammation

**DOI:** 10.3390/medicina62101939

**Published:** 2026-10-08

**Authors:** Carmen Lara-Apolinario, Cesar Martinez-Carral, Desiree Fischetti, Adexe J. Fulgencio-González, Casimira Domínguez, Pedro C. Lara

**Affiliations:** 1 Preventive Medicine and Public Health Department, Infanta Elena University Hospital, 28342 Madrid, Spain; 2 Medicine Department, Fernando Pessoa Canarias University, 35450 Guía, Spain; 3 Preventive Medicine and Public Health Department, Cruces University Hospital, 48903 Barakaldo, Spain; 4 Laboratory Medicine Department, Dr Negrín University Hospital, 35010 Las Palmas de Gran Canaria, Spain; 5 Canarian Comprehensive Cancer Center, San Roque University Hospital, 35001 Las Palmas de Gran Canaria, Spain; 6 Canarian Institute of Cancer Research, 38320 La Laguna, Spain

**Keywords:** cannabis, cocaine, opioids, amphetamine/methamphetamine, immunoassay, combination, NLR

## Abstract

*Background and Objectives:* This study aims to compare, for the first time, the impact of different substances detected by immunoassay, either alone or in combination, in the values of neutrophil-to-lymphocyte ratio (NLR) as a marker of systemic inflammation in a large series of cases. *Materials and Methods:* Patients with immunoassay urine tests positive for at least one of the following substances of abuse: cannabis, cocaine, amphetamine or opioids, from January 2021 to December 2025 at the Doctor Negrín University Hospital Laboratory were included in this study. A complete blood count, including leukocyte differential count, performed at the time of the substance detection urine analysis, was mandatory. *Results*: A total of 5008 samples were included in the present study. Cannabis alone was detected in 1831 cases, showing lower NLR (median 2.39) than cocaine (1055 cases, median: 2.44), opioids (227 cases, median: 3.01) and amphetamine/methamphetamine (487 cases, median: 3.28) (*p* < 0.0001). The detection of combined substances increased the NLR (*p* < 0.0001) mainly in combinations including amphetamines. In a lineal regression analysis, age (*p* < 0.0001), sex (*p* = 0.012) and substance consumption pattern (*p* < 0.0001) were independent predictors of NLR values. *Conclusions*: Our study demonstrated for the first time that the inflammatory effects of cannabis, cocaine, opioids and amphetamine/methamphetamine differ significantly, with amphetamine/methamphetamine and opioids being related to the highest NLR values. We also analyzed, for the first time, in the largest patient series studied, how the concomitant detection of different patterns of substances of abuse affects systemic inflammation patterns. The first finding is that an increase in the number of substances detected increases the NLR values. The second finding is that combinations including amphetamine/methamphetamine showed the highest inflammation marker (NLR) values.

## 1. Introduction

Substance abuse constitutes a major global public health challenge due to its chronic and relapsing nature [1]. Addiction is therefore recognized as a chronic brain disorder characterized by compulsive substance use, intense drug craving, and a high propensity for relapse, even after prolonged periods of abstinence [2]. These behavioral manifestations result from profound dysregulation of motivational neurocircuitry, including alterations in dopaminergic and opioid signaling within the basal ganglia during the binge/intoxication phase, as well as the recruitment of stress-related neurotransmitter systems in the extended amygdala during withdrawal [3].

Beyond its effects on the central nervous system, addiction is increasingly recognized as a condition associated with widespread physiological disturbances. Among these, chronic low-grade systemic inflammation has emerged as an important contributor to both the development and persistence of substance dependence [4]. Elevated levels of inflammatory mediators may affect neuroimmune communication, synaptic plasticity, and clinical outcomes, suggesting that inflammatory processes may actively contribute to the progression of addictive disorders rather than merely represent a consequence of substance use [5].

The use of unregulated psychoactive substances represents an important trigger of both peripheral and central immune activation. Chronic exposure has been associated with significant alterations in several hematological parameters, including the neutrophil-to-lymphocyte ratio (NLR) and total white blood cell count [6,7,8]. These findings support the presence of persistent immune dysregulation among individuals with substance dependence.

The neutrophil-to-lymphocyte ratio (NLR) has attracted increasing attention because of its simplicity, low cost, and potential applicability in clinical practice. Although substantial evidence supports the presence of immune dysfunction and a pro-inflammatory state in individuals with substance abuse [6,7,8,9,10,11,12,13,14], studies specifically investigating NLR remain relatively scarce and have yielded heterogeneous results [5,6,7,8,9,10,11,14]. The magnitude and clinical relevance of these alterations appear to vary according to both the substance involved and the characteristics of the study population [5,15,16].

We recently reviewed the available evidence regarding the effects of substance abuse on NLR [17]. No clear evidence was identified for cannabis, while only one study was available for cocaine. In contrast, stronger evidence was found for amphetamine/methamphetamine and opioids [17]. Nevertheless, the existing literature evaluating NLR and related inflammatory biomarkers remains limited and is characterized by substantial methodological heterogeneity, which hampers comparisons across studies [18,19,20].

Furthermore, the lack of prospective longitudinal investigations and the absence of standardized clinical thresholds limit the translation of these biomarkers into routine clinical practice [7,18,19,20]. The identification and validation of reliable inflammatory markers, such as NLR, could potentially improve diagnostic assessment, patient stratification, prognostic evaluation, and treatment monitoring in addiction medicine. However, the current body of evidence remains insufficient to support definitive clinical recommendations [7,18,19,20].

Polysubstance use disorder (PSUD) is increasingly recognized and diagnosed, with characteristic patterns involving the concomitant use of multiple substances [21,22]. To date, only two studies have investigated the association between self-reported substance use disorders and systemic inflammatory markers in adults [23,24]. These studies included relatively small sample sizes (30 and 76 cases, respectively), and neither performed stratification according to specific substance combinations. Both studies reported higher NLR values in cases compared with controls [23,24].

To our knowledge, no studies have directly compared the effects of different substances of abuse on systemic inflammation as assessed by NLR, nor have they investigated the inflammatory potential associated with the different possible combinations of these substances.

The aim of the present study is to compare, for the first time, the impact of substances detected by immunoassay, either individually or in combination, on NLR values as a marker of systemic inflammation. This approach may help clarify the contribution of systemic inflammation, as reflected by NLR, to the pathophysiology of substance use disorders and explore its potential clinical value in decision-making, particularly in advancing personalized approaches to the diagnosis and management of addictive disorders.

## 2. Methods

### 2.1. Patients

Patients with immunoassay urine analyses positive for at least one of the following substances of abuse: cannabis, cocaine, amphetamine or opioids, from January 2021 to December 2025, at the Doctor Negrín University Hospital Laboratory, were included in this study. Patients with acute poisoning exclusively due to ethanol were excluded. A complete blood count, including leukocyte differential counts, performed at the time of the substance-detection urine analysis was mandatory.

Samples were treated anonymously, including only sex and date of birth. No information was therefore available about possible comorbidities or abuse of other substances.

### 2.2. Laboratory Procedures

Urine samples were analyzed for the presence of commonly abused substances using an automated Alinity C clinical chemistry system (Abbott Laboratories, Abbott Park, IL, USA). Drug screening was performed with homogeneous immunoassay methods employing the manufacturer’s validated reagent kits. The analytical panel comprised amphetamine/methamphetamine, cocaine (identified by detection of its principal metabolite, benzoylecgonine), cannabinoids (THC-COOH), and opiates. Results were interpreted qualitatively as positive or negative according to the assay cut-off values established by the manufacturer: 50 ng/mL for cannabinoids, 300 ng/mL for cocaine metabolites and opiates, and 500 ng/mL for amphetamines and methamphetamines.

Peripheral venous blood was collected at the time of each urine substance test. Therefore, the blood tests were performed without mandatory fasting periods. Hematological parameters, including total and differential leukocyte counts, were measured in EDTA-anticoagulated whole blood using a CELL-DYN Sapphire hematology analyzer (Abbott Diagnostics, Abbott Park, IL, USA). This instrument integrates electrical impedance, flow cytometry, and optical light-scattering techniques to perform automated blood cell analysis. The neutrophil-to-lymphocyte ratio (NLR) was subsequently determined by calculating the quotient of the absolute neutrophil count and the absolute lymphocyte count.

### 2.3. Ethics

The study was performed in compliance with the ethical standards established in the Declaration of Helsinki. Approval was first obtained from the Ethics Committee of Fernando Pessoa University on 24 June 2024 (Protocol No. PI 005-2024), followed by authorization from the Ethics Committee of Doctor Negrín University Hospital on 26 February 2026 (Protocol No. 2026-082-1). Given the retrospective design of the study and the exclusive use of previously collected, fully anonymized biological specimens, both ethics committees determined that informed consent was not required. Accordingly, no written informed consent was requested or obtained from the study participants.

### 2.4. Statistical Analysis

The normality of continuous variables was assessed using the Kolmogorov–Smirnov test. Relationships between variables were examined by applying either Pearson’s or Spearman’s correlation analysis, depending on the distributional characteristics of the data and the type of variables involved. Differences in continuous variables according to sex or age category were evaluated using the statistical procedure most appropriate for each comparison, including Student’s *t*-test, Wilcoxon rank-sum test, or Kruskal–Wallis test.

Categorical data are expressed as absolute frequencies and percentages, whereas continuous variables are summarized as either mean values or median values with the corresponding interquartile range (IQR, 25th–75th percentile), according to their distribution. When the assumption of normality was not fulfilled, non-parametric methods were used, including rank-based correlation analyses for variables with non-normal distributions. A simple linear regression was used to analyze the relation of NLR with known data of age, sex and substances detected. Dependent variables were coded as follows: sex, (positive or negative) substance detection (one, two, three or four detected substances), and age (as a continuous variable). NLR was not log-transformed. Statistical significance was defined as a two-tailed *p* value below 0.05. All statistical analyses were carried out using IBM SPSS Statistics software (version 26; IBM Corp., Armonk, NY, USA).

## 3. Results

Five thousand and nineteen samples resulted positive for the substances studied between 1 January 2021 to 31 December 2025. No blood test was performed at the time of substance detection in 11 cases. A final number of 5008 samples was included in the present study. Mean age was 41.89 ± 15.21 years (range 16–95), median 41 y. There were 1412 women (28.2%) and 3596 men (71.8%).

Substances detected included cannabis, cocaine, opioids and amphetamine/methamphetamine either alone or in different combinations (Table 1). Cannabis and cocaine were the most frequently combined substances. Opioids are more frequently consumed in combinations (394 cases) than alone (227 cases) (Table 1).

**Figure 1:**
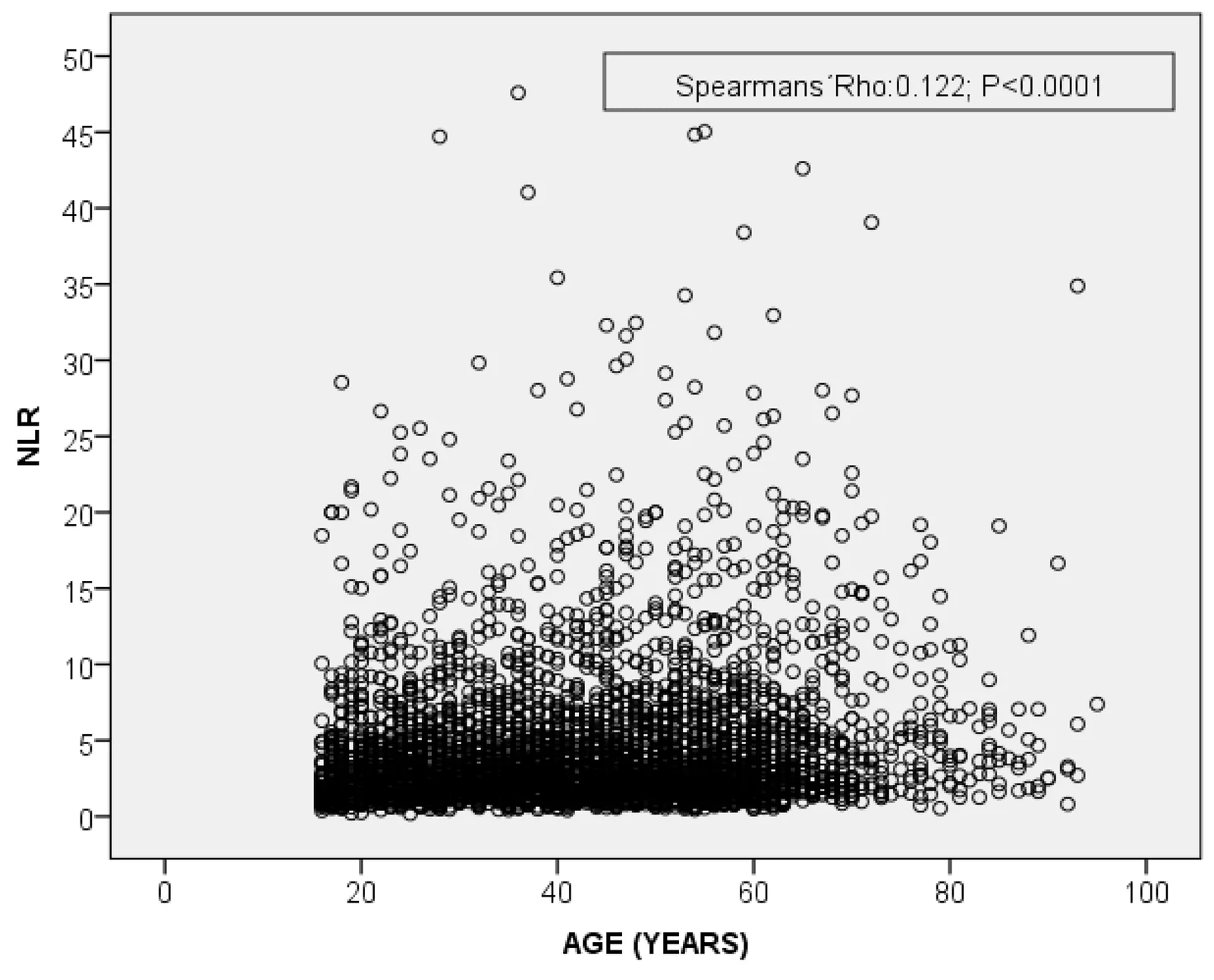
Relation between NLR and age.

The NLR mean of the whole series was 4.02 ± 4.55, median 2.58, Inter Quartile Range (IQR) 2.87. NLR values increased with age (*p* < 0.0001) (Figure 1).

There were also differences in NLR by sex. Women had lower NLR values (mean 3.75 ± 4.11; median 2.49 IQR 2.42) than men (4.13 ± 4.71; median 2.63 IQR 3.08) (*p* < 0.0001).

**Figure 2:**
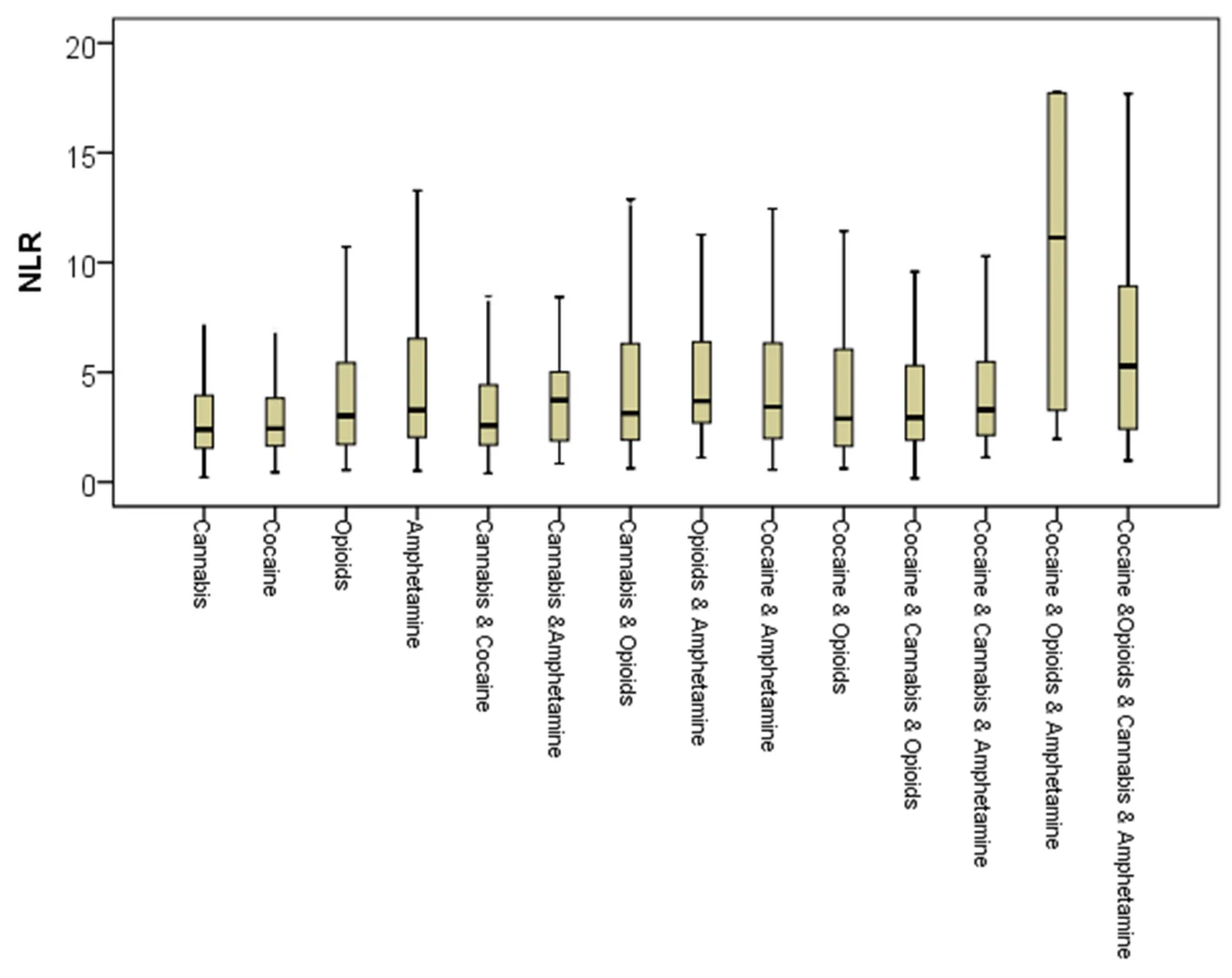
NLR and substance detection.

Cannabis showed lower NLR values (1831 cases, median 2.39) than cocaine (1055 cases, median 2.44), opioids (227 cases, median 3.01) and amphetamine/methamphetamine (487 cases, median 3.28) (*p* < 0.0001). Combined substance detection increased the NLR (Table 1) (Figure 2), mainly in combinations including amphetamines.

**Figure 3:**
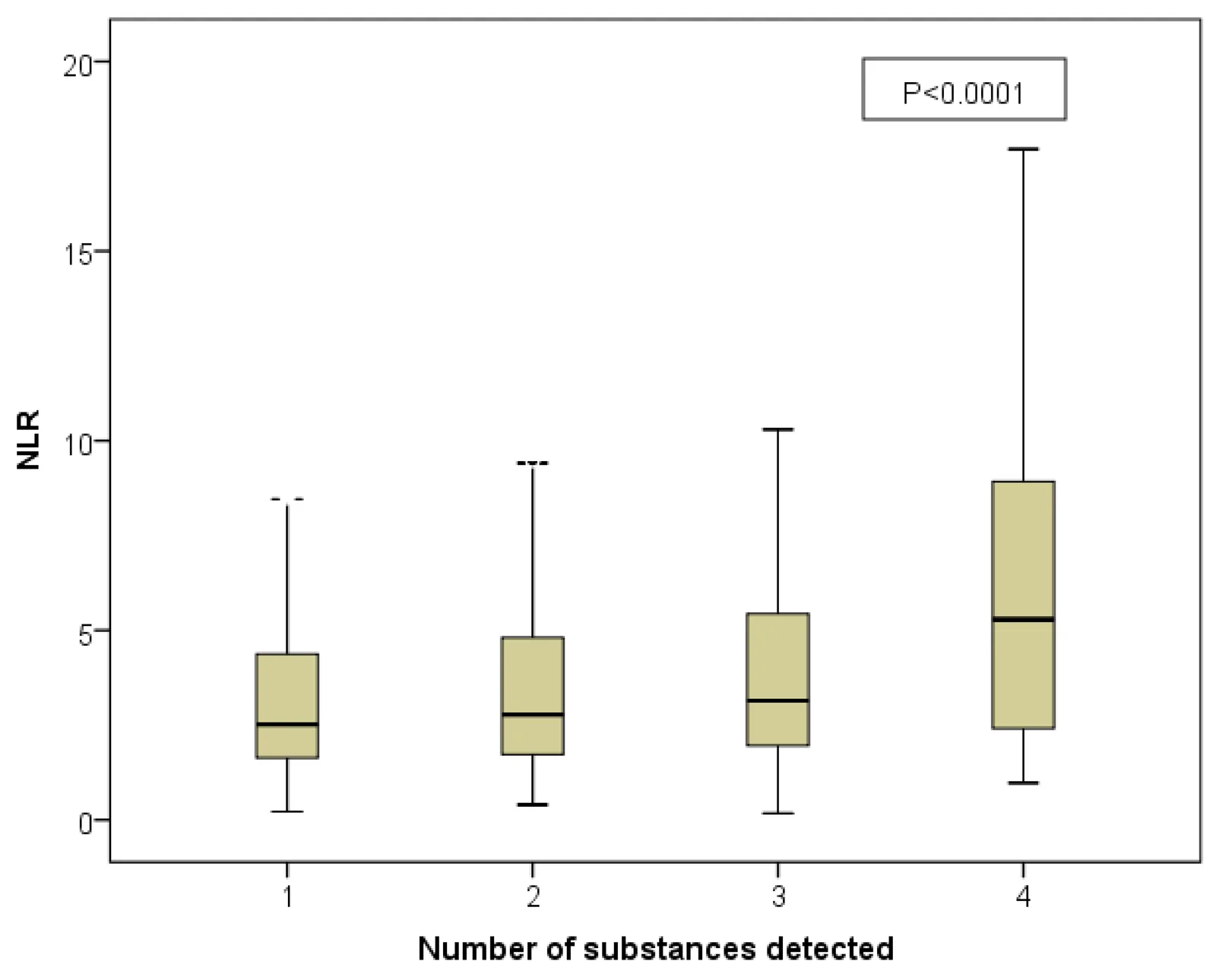
Relation between the number of detected substances and NLR.

Pooled analysis of the cases with one, two, three or four substances detected showed a significant increase in NLR with increasing numbers of substances detected (Table 2) (Figure 3).

In fact, cases with one drug detected showed a lower NLR (median 2.51) compared to those with two drugs (median 2.77) and those with three (3.14) or four substances (5.28) (*p* < 0.0001). When the low number of cases in some groups of combined detected substances is excluded, the overall gradient is unchanged.

A simple lineal regression analysis was performed including age, sex and number of substances detected as possible predictors of NLR values. Age (β: 0.041 (95% CI: 0.033–0.050; T: 9.868; *p* < 0.0001), sex (β: 0.35 (95% CI: 0.076–0.633); T: 2.494; *p* = 0.013) and substances detected (β: 0.381 (95% CI: 0.157–0.604); T: 3.338; *p* < 0.0001), were independently related to the NLR values. Model’s R^2^ was 0.22. Comparison between substance types was unadjusted.

## 4. Discussion

Our study analyzed, for the first time, in the largest series of cases ever reported, the comparison of NLR values in patients detected with cannabis, cocaine, opioids and amphetamine/methamphetamine either alone or in combination.

Our results found increased levels of NLR values associated with the detection of cannabis followed by cocaine and opioids. Amphetamine/methamphetamine showed the highest values of NLR detected (*p* < 0.0001). When concomitant detection of these substances was analyzed, the increasing number of substances detected increased the NLR values. Interestingly, combinations which included amphetamine/methamphetamine showed the highest NLR values.

No previous comparative evidence is available on the effect of different substances in the NLR values nor on the role of concomitant detection of substances in NLR. The reported data on cannabis were contradictory regarding the role of this substance in increasing NLR [16]. The relationship between cannabis use and systemic inflammation is complex, showing a discrepancy between preclinical mechanisms and clinical findings. Mechanistically, cannabinoids exert immunomodulatory and anti-inflammatory effects primarily through the activation of the CB2 receptor (CB2R), which is expressed in immune cells and microglia within the central nervous system [25,26]. However, clinical evidence is more heterogeneous. In patients with schizophrenia, acute cannabis use has been associated with lower inflammatory markers such as C-reactive protein (CRP), whereas cannabis cessation increases these markers [27]. In contrast, synthetic cannabinoids are associated with pro-inflammatory hematological changes, including leukocytosis, neutrophilia, and elevated monocyte counts [28]. Moreover, population-based studies have found no significant differences in systemic inflammatory markers, including hs-CRP and the neutrophil-to-lymphocyte ratio (NLR) between regular marijuana users and never-users after adjustment for confounding factors [29]. Overall, the clinical inflammatory effects of cannabis would depend on factors such as cannabinoid composition, psychiatric status, and individual characteristics [27,28,29].

The only available study on cocaine demonstrated a clear increase in NLR [30]. Mechanistically, this increase in the neutrophil count relative to lymphocytes reflects cocaine’s broad modulation of innate and adaptive immune pathways. Cocaine acts as an agonist for toll-like receptor 4 (TLR4) signaling, triggering downstream pro-inflammatory cascades [31]. Furthermore, chronic exposure shifts systemic cellular immunity by altering T-cell subset distributions and circulating cytokine profiles [32], which can directly disrupt peripheral leukocyte homeostasis and promote sustained neutrophil recruitment.

**Figure 4:**
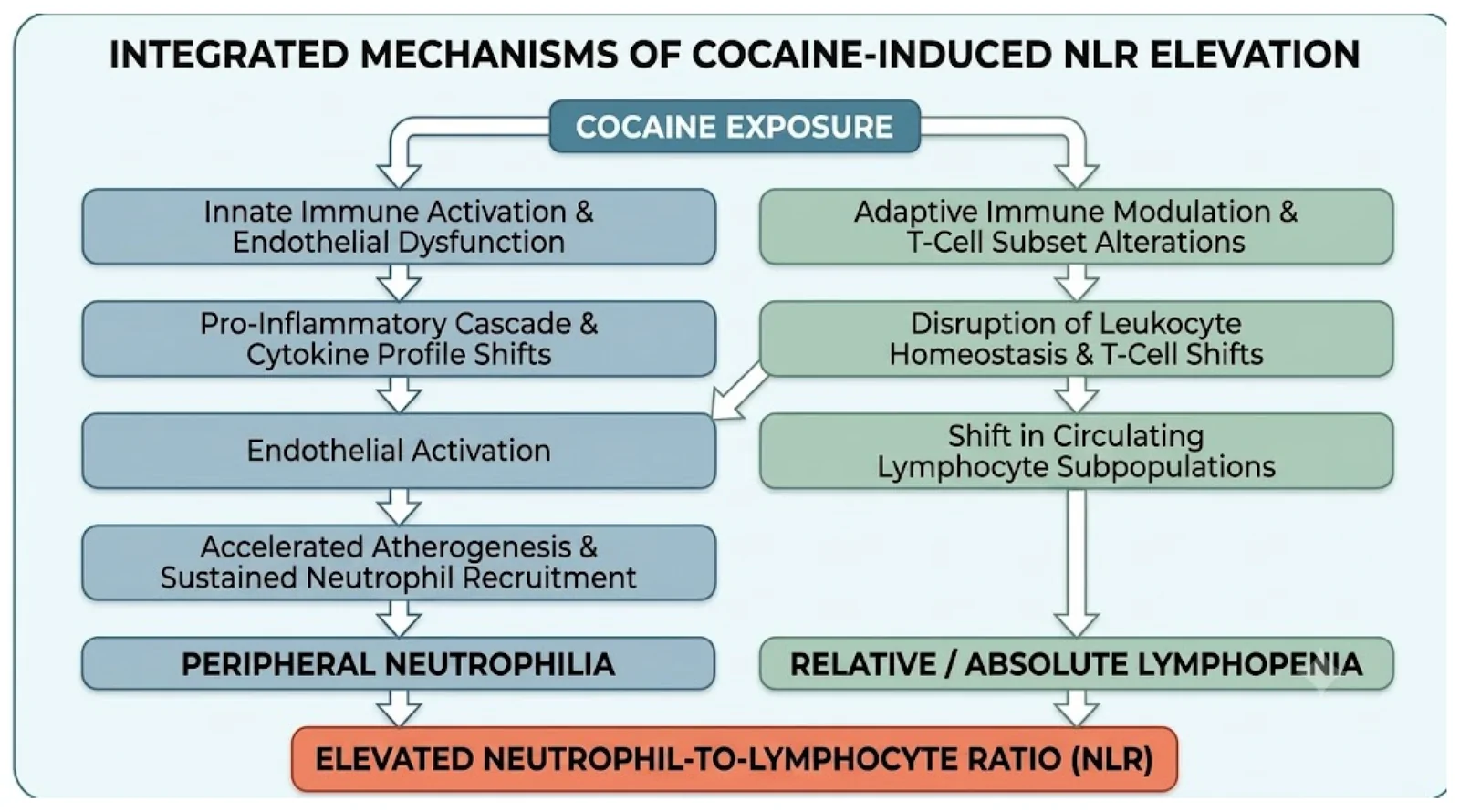
Integrated mechanisms of cocaine-induced NLR elevation.

At the tissue and cellular level, this elevated inflammatory state interacts closely with vascular and microvascular dysfunction. Cocaine exposure induces endothelial activation and upregulates adhesion molecules, driving systemic vascular inflammation and accelerating atherogenesis [33]. Concurrently, cocaine compromises cellular homeostasis by inducing endoplasmic reticulum stress, dysregulating autophagy, and breaching vascular barrier integrity [34]. Taken together, the elevation in NLR observed in our study, may reflect the peripheral manifestation of these convergent pathways, where cocaine-induced innate immune activation [31], lymphocyte subtype alterations [32], and end-organ vascular inflammation [33,34] reinforce a sustained systemic pro-inflammatory profile (Figure 4).

In contrast, there is more evidence of the increase in the NLR induced by opioids and methamphetamine [17].

**Figure 5:**
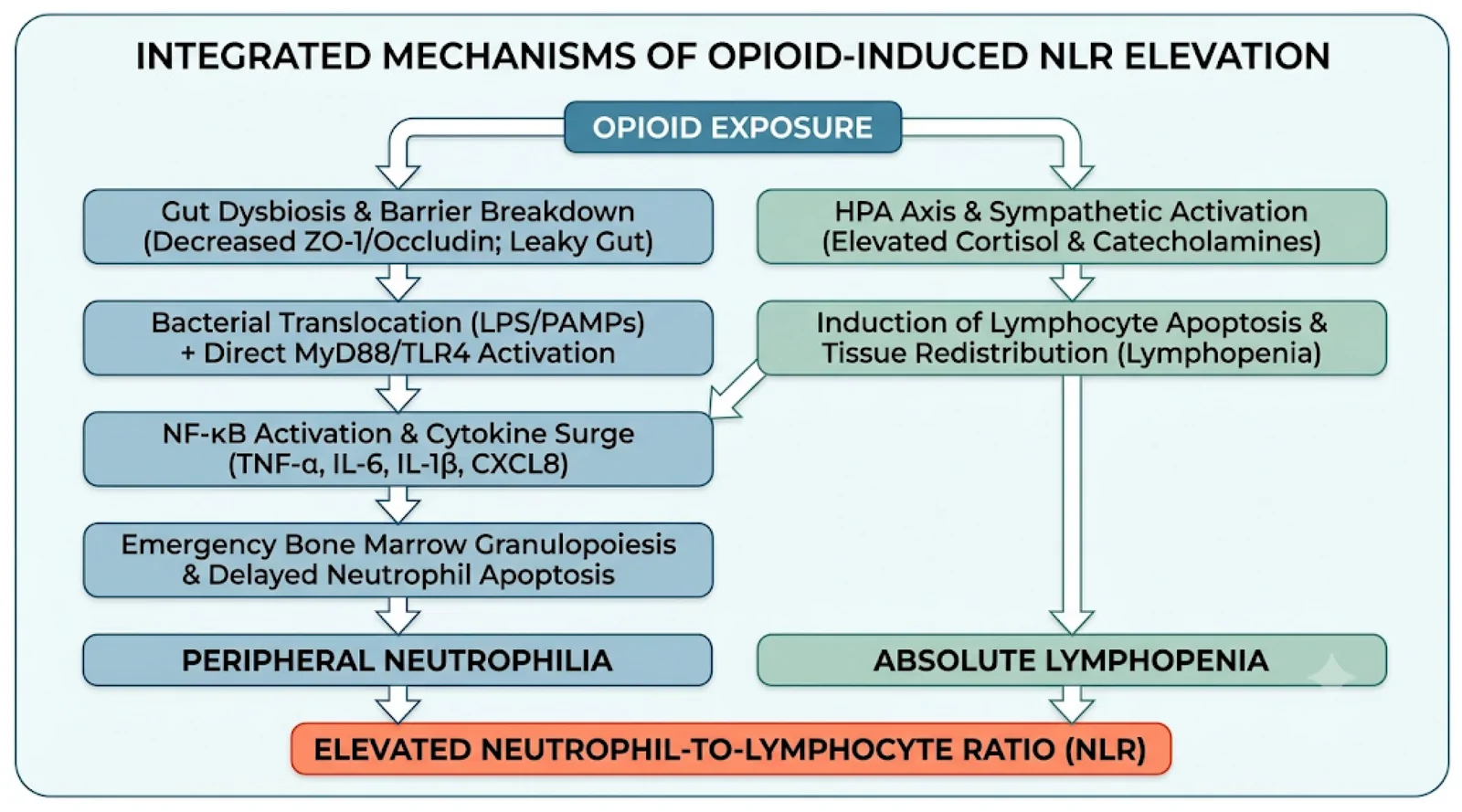
Integrated mechanisms of opioid-induced NLR elevation.

Our findings showed higher NLR values in cases of detected opioids. This imbalance would result from chronic opioid-induced immune activation through neuroimmune, gut–barrier, and neuroendocrine mechanisms (Figure 5).

One major pathway involves the activation of the toll-like receptor 4 (TLR4) on monocytes, macrophages, and microglia, independent of classical μ-opioid receptor signaling. TLR4 activation induces NF-κB signaling and the release of pro-inflammatory cytokines (TNF-α, IL-1, IL-6, and CXCL8), promoting emergency granulopoiesis, neutrophil recruitment, and delayed neutrophil apoptosis, thereby sustaining systemic inflammation [6,20,35,36,37,38,39].

Opioids also disrupt intestinal homeostasis by impairing gut motility, altering the microbiota, and reducing epithelial tight junction integrity, leading to increased intestinal permeability (“leaky gut”) [40,41]. This facilitates the translocation of bacterial products such as lipopolysaccharide (LPS), which chronically stimulates TLR4 signaling and maintains elevated inflammatory markers, including sCD14, LBP, and hs-CRP [6,20,35].

At the same time, opioids suppress adaptive immunity by activating the hypothalamic–pituitary–adrenal axis and sympathetic nervous system, increasing cortisol and catecholamine release. These neuroendocrine changes induce T-cell apoptosis and lymphopenia, particularly affecting CD4+ and CD8+ T cells, while expanding innate myeloid populations, thereby directly contributing to NLR elevation [35,42,43,44].

Recent studies further indicate that opioids reshape immune phenotypes and metabolism by promoting immune activation, T-cell senescence, and metabolic dysfunction linked to systemic inflammation [37,43,45]. Clinically, opioid-induced increases in NLR have been associated with reduced immunotherapy efficacy and poorer survival in patients with non-small cell lung cancer, highlighting the prognostic and therapeutic relevance of opioid-driven systemic inflammation [46].

**Figure 6:**
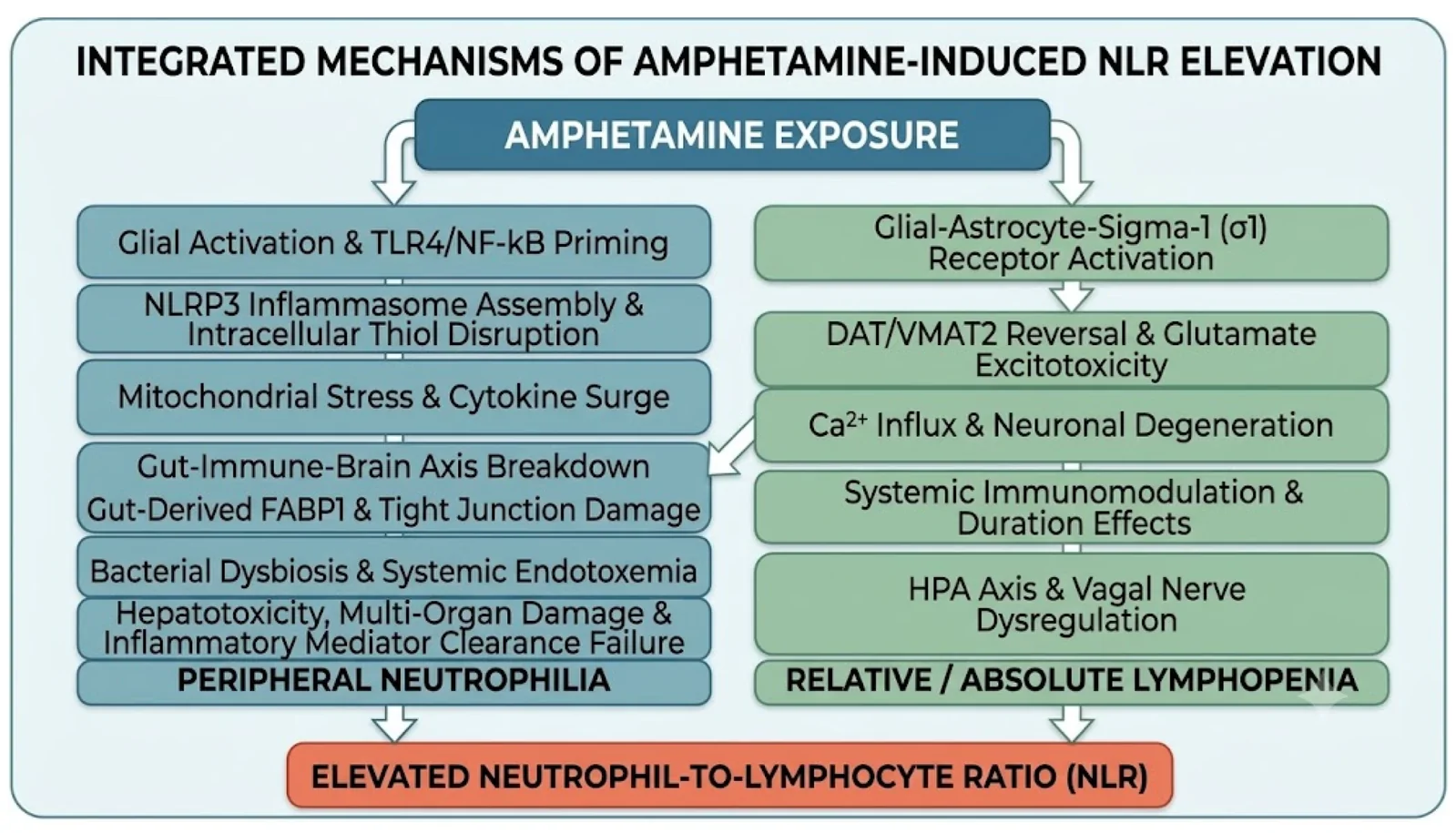
Integrated mechanisms of amphetamine/methamphetamine-induced NLR elevation.

Amphetamine/methamphetamine detection in our study was related to the highest NLR values. Amphetamine exposure promotes systemic and central inflammation through interconnected neuroimmune, cellular, and molecular mechanisms, leading to elevated inflammatory markers and increased neutrophil-to-lymphocyte ratio (NLR) [47,48,49] (Figure 6).

A key mechanism is the direct activation of microglia through the TLR4/MD-2 complex and astrocytes through Sigma-1 receptors, triggering intracellular inflammatory signaling, oxidative stress, and the release of pro-inflammatory cytokines, including IL-6, IL-1β, TNF-α, IL-18, and IL-23 [47,49]. Methamphetamine (METH)-induced dopamine and glutamate dysregulation further amplifies astrocytic NF-κB activation, neurotoxicity, and chronic microglial activation, while elevated peripheral IL-6 is associated with altered brain functional connectivity [47,48].

Inflammation is further intensified through the activation of the NLRP3 inflammasome. Methamphetamine primes NF-κB-dependent expression of NLRP3 and pro-IL-1β and subsequently induces inflammasome assembly through mitochondrial reactive oxygen species (mROS) production and lysosomal Cathepsin B release, leading to Caspase-1 activation and the maturation of IL-1β and IL-18, which amplify downstream inflammatory cytokine production [50,51]. Pharmacological inhibition of NLRP3 markedly reduces tissue injury and inflammatory responses, highlighting its central role [50].

Methamphetamine also disrupts the gut–immune–brain axis by damaging intestinal epithelial tight junctions, promoting dysbiosis and lipopolysaccharide (LPS) translocation into the circulation, while simultaneously impairing blood–brain barrier integrity through the loss of tight junction proteins and astrocytic dysfunction [48,49,52]. These alterations facilitate the entry of peripheral inflammatory mediators into the brain, further enhancing microglial activation and neuroinflammation. Additional contributions from hepatotoxicity, splenomegaly, and hypothalamic–pituitary–adrenal axis activation further sustain chronic systemic and central immune activation [49].

Overall, amphetamine-induced inflammation results from the coordinated activation of glial TLR4 signaling, glutamate excitotoxicity, NLRP3 inflammasome activation, and disruption of the gut–brain and blood-brain barriers, providing a mechanistic basis for the elevated systemic inflammatory markers and NLR observed in amphetamine exposure [40,47,48,49,50,51,52].

Our results also showed that the concomitant detection of different patterns of substance abuse affects systemic inflammation patterns. First, an increase in number of substances detected increases the NLR values; second, combinations including amphetamine/methamphetamine showed the highest NLR values. We should also acknowledge that simultaneous positivity may also reflect greater intensity or chronicity of use, alongside the pharmacological potentiation we propose here.

**Figure 7:**
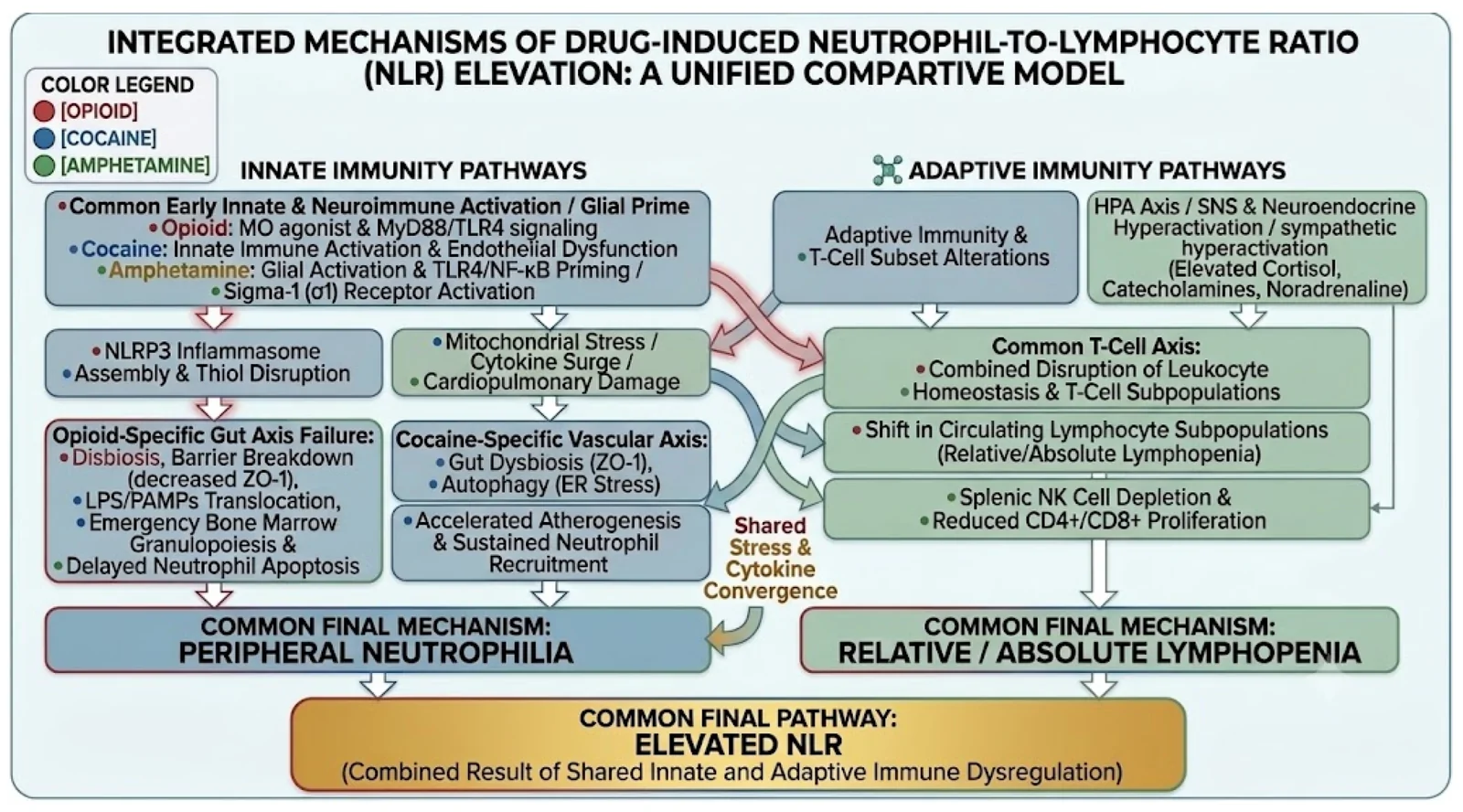
Integrated mechanisms of cocaine-, opioid- and amphetamine/methamphetamine-induced NLR elevation.

These clinical results need further biological studies to elucidate the characteristics of this polysubstance potentiation of inflammation as estimated by NLR values. According to the available knowledge, summarized above, some of the substances seem to share similar mechanisms leading to inflammation (Figure 7).

The elevated neutrophil-to-lymphocyte ratio (NLR) observed across opioid, amphetamine, and cocaine exposure reflects a convergent systemic inflammatory state driven by overlapping innate immune hyperactivation and neuroendocrine-mediated adaptive suppression. Mechanistically, all three substance classes converge on toll-like receptor 4 (TLR4) signaling to drive acute-phase innate immune cascades. Opioids act as non-stereoselective agonists or allosteric modulators of the myeloid differentiation protein 2 (MD-2)/TLR4 complex on monocytes, macrophages, and microglia [35,36], amphetamines trigger direct agonism on microglial TLR4/MD-2 complexes alongside Sigma-1 receptor activation [49] and cocaine acts as an agonist for TLR4 signaling [31]. Downstream activation of the MyD88 and NF-κB pathways stimulates the systemic surge of canonical pro-inflammatory cytokines—including TNF-α, IL-1β, IL-6, and CXCL8—which accelerates emergency bone marrow granulopoiesis, mobilizes mature neutrophils, and impairs physiological neutrophil apoptosis, thereby establishing sustained peripheral neutrophilia [6,20,38,39,48,49,50,53,54,55].

This peripheral innate activation is significantly reinforced by structural barrier breakdowns along the gut–immune–brain and vascular axes. Both opioids and amphetamines induce severe gastrointestinal dysbiosis, gut ischemia, and mucosal architecture failure marked by the downregulation of epithelial tight junction proteins such as ZO-1, occludin, and claudin-1 [40,41,49,52]. This gut hyperpermeability promotes the systemic translocation of lipopolysaccharide (LPS) and pathogen-associated molecular patterns (PAMPs), sustaining low-grade chronic endotoxemia and systemic inflammatory tone [35,52,54]. Concurrently, amphetamine and cocaine exposure exacerbate systemic and central organ damage: cocaine drives endothelial activation, upregulates adhesion molecules, dysregulates autophagy, and induces endoplasmic reticulum stress to breach vascular barrier integrity [33,34], while amphetamines trigger NLRP3 inflammasome assembly via mitochondrial ROS, lysosomal Cathepsin B leakage, and neurotransmitter excitotoxicity [48,50,51,55].

Simultaneously, all three drug categories drive relative or absolute lymphopenia through neuroendocrine hyperactivation and direct T-cell modulation, creating the cellular divergence that inflates the NLR. Opioids and amphetamines hyperactivate the hypothalamic–pituitary–adrenal (HPA) axis and sympathetic nervous system (SNS), inducing a surge of systemic glucocorticoids (cortisol) and catecholamines [35,42,49]. Cortisol upregulates pro-apoptotic signals (Bax, caspase-3) while downregulating anti-apoptotic markers (Bcl-2), triggering widespread apoptosis, depletion, and compartmental redistribution of naïve and memory CD4+ and CD8+ T cells [43,44]. Similarly, cocaine shifts adaptive immunity by altering T-cell subset distributions and Th1/Th2/Th17 cytokine profiles [32], and chronic amphetamine exposure leads to reduced CD4+/CD8+ T-cell proliferation and splenic NK cell depletion [56].

We can suggest that, when concomitant detection of several substances is observed, the elevation of NLR serves as a unified peripheral biomarker capturing the combined impact of shared TLR4/NF-κB innate priming, gut/vascular barrier breach, and neuroendocrine-driven lymphopenia across substance use disorders. Collectively, these convergent mechanisms sustain chronic elevations of inflammatory mediators and may contribute to cardiovascular disease, neuroinflammation, immune dysfunction, and accelerated biological aging associated with substance use disorders.

Limitations: This study has several limitations. No clinical data is related to the substance detection; therefore no information was available about possible comorbidities or the abuse of other substances. The indication for testing may itself vary across substances and could therefore contribute to the observed gradient in NLR independently of their pharmacological effects. Non-clinically detected ethanol co-use may be present in our patient’s series. Ethanol has a recognized inflammatory effect specially when combined with cocaine, in view of cocaethylene formation when the two are co-used. Only cannabis, cocaine, amphetamine/methamphetamine and opioids were included in the study. The immunoassay has important limitations as it can yield false-positives and false-negatives. Finally, the low number of cases in some groups of combined detected substances, can preclude the reproducibility of the present results.

## 5. Conclusions

Our study demonstrated for the first time that the inflammatory effects of cannabis, cocaine, opioids and amphetamine/methamphetamine differ significantly, with amphetamine/methamphetamine and opioids being related to the highest NLR values.

We analyzed for the first time, in the largest patient series studied by immunoassay, how the concomitant detection of different patterns of substances of abuse affects systemic inflammation patterns. The first finding is that the increasing number of substances detected increases the NLR values. The second finding is that combinations including amphetamine/methamphetamine showed the highest inflammation marker (NLR) values. A strong biological basis, mainly through neuroimmune, gut–barrier, and neuroendocrine mechanisms, supports this inflammatory effect. These convergent mechanisms that increase systemic inflammation may contribute to cardiovascular disease, neuroinflammation, immune dysfunction, and accelerated biological aging associated with substance use disorders. Future studies would be prospectively designed to include a detailed clinical context at the time of sampling. Furthermore, the lack of prospective longitudinal investigations and the absence of standardized clinical thresholds limit the translation of these biomarkers into routine clinical practice [7,18,19,20]. Establishing reliable inflammatory markers such as NLR could improve diagnostic assessment, patient stratification, prognostic evaluation, and treatment monitoring in addiction medicine.

## Figures and Tables

**Table 1:** Substances detected by immunoassay and NLR.

	Number of Cases	NLR	
One Drug Detected		Mean ± SD	Median (IQR)
Cannabis	1831	3.53 ± 3.78	2.39 (2.40)
Cocaine	1055	3.56 ± 3.51	2.44 (2.19)
Opioids	227	4.81 ± 5.62	3.01 (3.74)
Amphetamine	487	5.71 ± 7.47	3.28 (4.50)
Two Drugs Detected			
Cannabis and Cocaine	769	3.81 ± 3.81	2.56 (2.75)
Cocaine and Opioids	152	5.08 ± 5.67	2.89 (4.41)
Cannabis and Opioids	77	5.4 ± 6.15	3.12 (4.61)
Cocaine and Amphetamine	80	4.50 ± 3.71	3.42 (4.38)
Opioids and Amphetamine	22	4.95 ± 3.37	3.68 (3.99)
Cannabis and Amphetamine	94	4.46 ± 3.69	3.73 (3.15)
Three Drugs Detected			
Cocaine and Cannabis and Opioids	127	4.76 ± 5.76	2.93 (3.54)
Cocaine and Cannabis and Amphetamine	71	4.63 ± 3.89	3.28 (3.49)
Opioids and Cocaine and Amphetamine	4	10.49 ± 8.41	11.12 (15.13)
Four Drugs Detected			
Opioids and Cocaine and Cannabis and Amphetamine	12	6.41 ± 4.96	5.28 (6.63)

**Table 2:** Number of substances detected and NLR values.

Number of Substances Detected	Mean ± SD	Median (IQR)
One	3.92 ± 4.58	2.51 (2.74)
Two	4.19 ± 4.28	2.77 (3.09)
Three	4.83 ± 5.27	3.14 (3.52)
Four	6.41 ± 4.02	5.28 (6.63)
TOTAL	4.02 ± 4.55	2.58 (2.87)

## Data Availability

Data is available upon request to the author of reference due to ethics limitations.
